# I rely on a little help from my friends: the effect of interpersonal and intrapersonal emotion regulation on the relationship between FOMO and problematic internet use

**DOI:** 10.1186/s12888-024-05834-9

**Published:** 2024-05-24

**Authors:** Mal Flack, William H Burton, Kim M Caudwell

**Affiliations:** 1 Researchers in Behavioural Addictions, Alcohol and Drugs, Charles Darwin University, Ellengowan Drive, Brinkin, NT Australia; 2 Discipline of Psychology, Faculty of Health, Charles Darwin University, Ellengowan Drive, Brinkin, NT Australia

**Keywords:** FOMO, Fear of missing out, Problematic social media use, PSMU, Doomscrolling, Emotion regulation, Intrapersonal emotion regulation, Interpersonal emotion regulation, Social media use, Problematic internet use

## Abstract

**Background:**

This study investigated the role of emotion regulation in relation to the links between fear of missing out (i.e., FOMO) and two components of problematic internet use: problematic social media use and doomscrolling.

**Methods:**

Participants (*N* = 603, *M*_age_ = 30.41, *SD*_age_ = 7.64; 49.1% male-identifying) completed measures of fear of missing out, intrapersonal and interpersonal emotion regulation, and problematic social media use, and doomscrolling. A parallel mediation model was tested to examine the nature of the associations between fear of missing out, intrapersonal, and interpersonal emotion regulation, in accounting for variance in the outcome measures.

**Results:**

Analyses revealed that the effect of fear of missing out on problematic social media use was fully mediated by both intrapersonal and interpersonal emotion regulation. In contrast, the effect on doomscrolling was fully mediated by intrapersonal emotion regulation only.

**Conclusions:**

Findings clarify the role of emotion regulation in explaining the relationship between fear of missing out and two types of problematic internet use, indicating a need to consider individual differences in emotion regulation in an evolving social media landscape.

**Supplementary Information:**

The online version contains supplementary material available at 10.1186/s12888-024-05834-9.

## Introduction

As internet use has expanded in terms of reach and behaviour, research has focused on types of problematic internet use. Since its inception, there has been extensive investigation in relation to the effects of social media use on human behaviour, with a considerable focus on concerns around potential negative impacts on health and wellbeing [1, 2]. One notable correlate of the various manifestations of problematic internet use throughout the research literature is known as the *fear of missing out* (FOMO) – a “pervasive apprehension that others might be having rewarding experiences from which one is absent” (3; p. 1841). FOMO is characterized by the desire to stay continually connected with what others are doing, with social media platforms serving as an efficient means to maintain a “continual connection with what is going on” (p. 1841). True to its nature, FOMO has great relevance to social media, with meta-analytic evidence indicating relationships between FOMO and general social network use, and yet, stronger relationships with problematic social media use (PSMU) [4]. Similarly, FOMO shows consistent correlations with increased depression and anxiety, fear of negative evaluation, and neuroticism, across various studies [4]. Based on this literature, it appears FOMO has particular relevance as a precipitant underlying PSMU.

The growth of social media platforms, users, and time spent using social media in recent years is considerable, with the typical social media user engaging in use across 6 or more social media platforms, for 2 h and 25 min per a day on average [5]. Where social media use is excessive and causes difficulties across domains of functioning, it is termed problematic media use (PSMU), and exhibits similarities with behavioral addictions more broadly [6]. Indeed, research findings related to PSMU and problematic internet use largely mirror those found in the area of substance addiction [6], with PSMU showing links with various psychopathologies and negative health outcomes, such as depression [7], Attention Deficit Hyperactivity Disorder (ADHD), anxiety, and sleep disruption [8]. Moretta et al. [6] have advocated for problematic internet use research to consider constructs that are better able to differentiate it from other behavioural addictions, or affective disorders. Indeed, given the scale of social media use, preponderance of social media platforms, and time spent using social media, a more nuanced understanding of PSMU is paramount.

A recent behaviour of interest in relation to the increased proliferation of smartphones and social media platforms is *doomscrolling*, referring to the consuming of excessive amounts of negative or upsetting online news [9]. Doomscrolling appears to have emerged in tandem with a broader line of research enquiry around the psychological impacts of COVID-19-related lockdowns, which are thought to have increased internet-mediated problematic behaviours (e.g., 10, 11, 12). Two studies so far have shown doomscrolling correlates with PSMU, psychological distress, and self-control - and also FOMO [13, 14]. While the former links are somewhat reconcilable within the broader addiction literature, the link between FOMO and doomscrolling appears a promising avenue for exploration within the present problematic internet use context, given an individual may experience FOMO when an event is considered taking place in the digital space. For instance, Price et al. [15] refer to Twitter posts where use of the term “doomscrolling” peaks, noting this occurred following the murder of George Floyd, after the 2020 US Presidential election, and during the storming of the Capitol on January 6 2021. As such, it appears social media users exhibit and express a need to stay engaged and connected during these events so as not to ‘miss out’, a consequence of engaging in a more “liquid society” where psychological processes are impacted by uncertainty and unpredictability [16]. In a similar vein, more qualitative research on FOMO has unveiled an emerging conceptual complexity, that incorporates in part a fear of missing timely interactions, and the opportunity to know others’ impressions [17]. This would indicate a need to stay online and engaged with sensational current events that tend to dominate mobile and online news platforms in comparison to traditional print media (see [18]). As such, PSMU and doomscrolling are both important outcome variables that warrant exploration in relation to FOMO.

## Emotion regulation

While links between FOMO and PSMU, and FOMO and doomscrolling have been observed, research on the potential mechanisms that explain how FOMO and other correlates influence problematic internet use is beginning to accumulate. The original conceptualization of FOMO by Przybylski et al. [3] indicates that FOMO is at its core a negative emotional state resulting from unfulfilled or dissatisfied needs. Pertinent to improving our understanding of the relationship between FOMO and aspects of internet addiction is therefore considering individual’s attempt to manage the ‘fear’, or negative emotional state associated with FOMO (i.e., emotion regulation). Emotion regulation is conceptualized as actions that are intended to manage heightened arousal that accompanies distress [19], with Gratz and Roemer [20] conceptualizing emotion dysregulation as the absence of or difficulties with adaptive emotion regulation. Difficulty with emotion regulation is relevant transdiagnostically, implicated in general psychopathology [21, 22], as well as substance and behavioural addictions [23, 24], including PSMU [25]. As such, emotion regulation may be a key explanatory intermediary between FOMO and engagement in PSMU. For instance, if an individual experiences FOMO-related distress, and they are experiencing difficulty regulating this distress, they may turn to problematic forms of internet use such as PSMU and doomscrolling in an attempt to alleviate that distress [26].

An important point with regard to emotion regulation, as Hofmann et al. [27] note, that the mainstay of research on the construct focuses on the intrapersonal (i.e., emotion dysregulation), rather than considering more interpersonal elements (i.e., interpersonal emotion regulation). While the former relates to managing emotional discomfort through internal processes or resources, the latter type necessitates an external entity such as a friend or confidant through which regulation is facilitated [27]. Accordingly, difficulties with intrapersonal emotion regulation when experiencing negative affect may lead individuals to rely on others to regulate their emotions, with difficulties in interpersonal emotion regulation linked to anxious attachment, trait and social anxiety, and depression [27, 28]. Focusing on both intrapersonal and interpersonal regulation appears especially relevant to understanding the relationship between FOMO and forms of problematic social media use, given the broad interpersonal space within which social media is situated.

## The present study

**Fig. 1:**
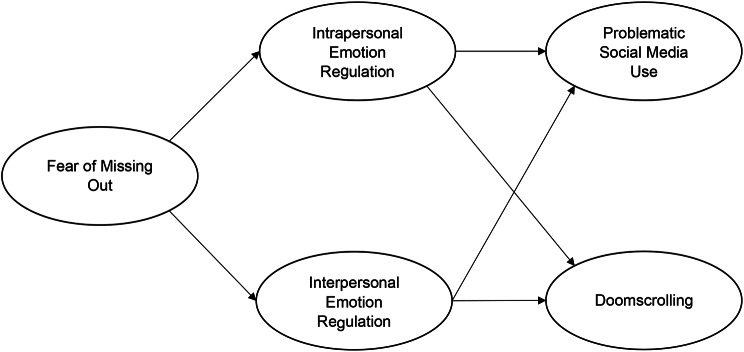
The hypothesised parallel mediation model

The available literature indicates that while problematic social media use is linked to a range of negative outcomes, less is known about the processes by which individuals find themselves engaging in problematic social media use. Similarly, the recent phenomenon of doomscrolling warrants further investigation within the broader realm of problematic internet use behaviours. Additionally, both intrapersonal and interpersonal emotion regulation appear to be potential mechanisms that can better account for how FOMO may lead to problematic use outcomes. The present study therefore aimed to test a hypothesized parallel mediation model whereby the effect of FOMO on both PSMU and doomscrolling was fully mediated by both intrapersonal emotion regulation, and interpersonal emotion regulation (see Fig. 1).

Specifically, it was hypothesized that: (1) FOMO would be positively associated with PSMU and Doomscrolling; (2) FOMO would be positively associated with both intrapersonal emotion regulation and interpersonal emotion regulation;(3) intrapersonal and interpersonal emotion regulation would be associated with PSMU and Doomscrolling, and; (4) the effect of FOMO on PSMU and Doomscrolling would be fully mediated by intrapersonal and interpersonal emotion regulation.

## Methods

### Data collection

Data were collected via an online survey created using Qualtrics, which was advertised as a part of a study on social media use via social media platforms (e.g., Facebook, Instagram, reddit). Participation was incentivized by use of a prize draw, where participants could opt in to win one of four AUD $25 gift cards on completion. In addition to the variables described in the below Measures section, the survey included demographic questions about age, gender, educational attainment, and employment. Data were collected between July and August 2023. An a-priori power analysis using the Monte Carlo Power Analysis by Schoemann et al. [29] for a parallel mediation model was conducted to determine the minimum sample size required to reach power of 0.80. Estimates of the effect size between the predictor, mediator, and outcome variables were set at *r* = .3 and the effect size between the mediators set at *r* = .6. The power analysis indicated a sample size of at least *n* = 470 was required. Ethical clearance was provided by the University Human Research Ethics Committee (H23038).

### Measures

#### Fear of missing out (FOMO)

FOMO was measured using the Fear of Missing Out Scale developed by Przybylski et al. [3]. The scale comprises 10 statements (e.g., “I fear others have more rewarding experiences than me”), requiring participants to indicate how true each one is of their everyday experience on a scale of 1 (“not at all true of me”) to 5 (“extremely true of me”), with higher total scores indicative of more extensive FOMO. The scale demonstrated good internal consistency (Cronbach’s α *=* 0.86).

#### Emotion regulation

The brief version of the Difficulties in Emotion Regulation scale (DERS-18) [30] was used to measure difficulties in emotion regulation. The DERS-18 includes six subscales, comprising 18 statements that refer to the experience or management of emotion, and a response scale ranging from 1 (*almost never*) to 5 (*almost always*). The subscales reflect (1) non-acceptance of emotional responses (*Non-Acceptance*; e.g., “When I’m upset, I become embarrassed for feeling that way”); (2) difficulty engaging in goal-directed behaviour (*Goals*; e.g., “…I have difficulty focusing on other things”); (3) impulse control difficulties (*Impulse*; e.g., “…I lose control over my behaviours”); (4) lack of emotional awareness (*Awareness*; e.g., “I pay attention to how I feel”); (5) limited access to emotion regulation strategies (*Strategies*; e.g., “…I believe that wallowing in it is all I can do”); and (6), lack of emotional clarity (*Clarity*; e.g., *I have no idea how I am feeling*). Noting that a total score can be used for the DERS [30], internal consistency for each subscale was acceptable, with Cronbach’s α values ranging from 0.61 (*Awareness*) to 0.82 (*Impulse*).

#### Interpersonal emotion regulation

The Interpersonal Emotion Regulation Questionnaire (IERQ) [27] was used to measure interpersonal emotion regulation. The 20-item scale comprises four factors, including *Enhancing Positive Affect* (e.g., “Because happiness is contagious, I seek out other people when I’m happy”), *Perspective-taking* (e.g., “When I am annoyed, others can soothe me by telling me not to worry”), *Soothing* (e.g., “When I feel sad, I seek out others for consolation”), and *Social Modelling* (e.g., “It makes me feel better to learn how others dealt with their emotions”), with participants indicating a response from 1 (“not true for me at all”) to 5 (“extremely true for me”). Internal consistency for these subscales was acceptable (i.e. Cronbach’s α values ranged from 0.78 to 0.82).

#### Problematic social media use

The Bergen Social Media Addiction Scale (BSMAS) [31] was used to measure PSMU. The unidimensional scale comprises six statements reflecting addictive elements of social media use (e.g., “How often during the last year have you tried to cut down on the use of social media without success?”), with participants responding on a scale from 1 (“very rarely”) to 5 (“very often”). Higher scores indicate more frequent problematic social media use. The scale demonstrated adequate internal consistency (Cronbach’s α *=* 0.79).

#### Doomscrolling

The Doomscrolling Scale [14] is a unidimensional scale comprising 15 statements (e.g., *I unconsciously check my newsfeeds for bad news*), for which participants provide a response from 1 (*strongly disagree*) to 7 (*strongly agree*). Scores ranged from 15 to 105, with higher scores indicating greater extent of doomscrolling behaviour. Cronbach’s α (0.96) indicated good internal consistency.

#### Social media usage

Social media habits were also collected, including hours per day spent on social media, social media sites used, and most preferred site. Sites offered to choose from were ‘Facebook’, ‘Snapchat’, ‘TikTok’, ‘Instagram’, ‘YouTube’, and ‘Twitter’, with the option to include other platforms.

### Analytic plan

IBM SPSS AMOS Version 28 was used to assess the proposed model shown in Fig. 1. across two stages. The first stage included the assessment of measurement model fit of the two mediators (DERS and IERQ). The two constructs of emotion regulation were examined and respecified as required to ensure their conceptual clarity and distinction as two separate, but related aspects of emotion regulation. A two-step approach was adopted to test the measurement model, whereby a subsample of participants was used for model calibration, with a hold-out sample reserved for cross validation. After confirming the suitability of the measurement model, the structural model was tested, where FOMO served as the predictor variable, and PMSU and doomscrolling as the outcome variables, given their noted conceptual and correlational overlap [14].

The Maximum Likelihood method was used to estimate the model parameters and we used several fit indices to assess model fit, including the Root Mean Square Error of Approximation (RMSEA) statistic as an index of absolute fit (RMSEA ≤ 0.08), and the Comparative Fit Index (CFI) and Tucker-Lewis Index (TLI) as measures of incremental (values ≥ 0.95). Given its sensitivity to mild departures from normality and larger sample sizes [32], the χ^2^ statistic was not used to assess model fit. Potential areas for model re-specification were identified by the model modification indices, standardized indicator loading (< 0.70), or non-significant regression paths, as well as high residual correlations. However, the re-specification of the models was guided firstly by theoretical considerations and existing literature.

## Results

### Participants

A total of 603 participants (*M*_age_ = 30.41, *SD*_age_ = 7.64; 49.1% male-identifying) provided informed consent and completed the online survey. The majority of participants reported having seen the study on Facebook (*n* = 183; 30.3%), followed by TikTok (*n* = 123; 20.4%), Instagram (*n* = 113; 18.7%), Twitter (*n* = 70; 11.6%), and LinkedIn (*n* = 35; 5.8%), with 80 (13.2%) reporting other means (e.g., word of mouth). In terms of educational attainment, the majority of participants held a Bachelor’s degree (*n* = 278; 46.1%), were pursuing or had completed a vocational qualification (*n* = 122; 20.2%), had completed all or some secondary education (*n* = 122; 20.2%), and were pursuing or had completed a postgraduate degree (*n* = 59; 9.8%). In terms of employment, the majority of the sample reported working full time (*n* = 339; 56.2%), followed by part time (*n* = 101; 34.6%), with 55 (9.1%) not currently working. In relation to social media platform usage, participants mostly reported use of Facebook (*n* = 352; 58.4%), followed by TikTok (*n* = 347; 57.5%), and Instagram (*n* = 299; 49.6%), with an average of 5.07 h a day spent using social media (SD = 4.80 h). Correlations and reliability coefficients for the model measures are included in Supplementary Table S1.

### Measurement model and calibration of DERS and IERQ

A random subsample of 300 participants was used for model calibration, with the hold-out sample comprising the remaining 303 participants.

Given intrapersonal and interpersonal emotion regulation abilities are different but related types of emotion regulation [27], the model fit of DERS and IERQ were first assessed independently as congeneric models [33] to ensure the acuity of each measure. Intrapersonal emotion regulation was modelled with each of the DERS subscale items (e.g., *Awareness*, *Clarity*, *Goals*, *Impulse*, *Non-acceptance* and *Strategies*) parceled into latent composite indicators. The full DERS model was a poor fit (i.e., RMSEA = 0.095; CFI = 0.963; TLI = 0.939). Inspection of the indictor loadings revealed *Awareness* and *Goals* loadings were below the adopted threshold of 0.70. Given recent research by Burton et al. [34] demonstrated that the *Awareness* and *Goals* subscales exhibit small, non-significant correlations with other emotion regulation scales, they were removed, and the model reassessed. The trimmed model incorporating the revised DERS measurement model exhibited good fit (i.e., RMSEA = 0.052; CFI = 0.997; TLI = 0.991).

Similar to the approach taken with the DERS, the IERQ items were parceled into their respective subscale indictors (i.e., *Enhancing Positive Affect*, *Soothing*, *Perspective*-*taking*, and *Social Modelling*). Fit for the IERQ calibration measurement model indicated the model required re-specification (i.e., RMSEA = 0.112; CFI = 0.984; TLI = 0.951). Inspection of the factor loadings revealed *Enhancing Positive Affect* was below the desired loading of 0.70 and the indicator was subsequently removed. As the model fit of the IER was unable to be further assessed in isolation due the limited degrees of freedom, the two emotion regulation factors were combined in one measurement model with the factors allowed to co-vary for further assessment. To ensure the uniqueness of each factor, indicators were permitted to only load on their respective factor. The fit estimates for the combined measurement model indicated the model was a sub-optimal fit to the data (RMSEA = 0.083; CFI = 0.972; TLI = 0.955). Inspection of modification indices revealed that *Perspective-taking* was correlated with two other indicators. Therefore, to enhance the conceptual clarity of the IERQ, the *Perspective-taking* subscales was removed, and the model retested. The full respecified measurement model incorporating both intrapersonal and interpersonal regulation, exhibited good fit in the calibration sample (RMSEA = 0.033; CFI = 0.996; TLI = 0.993). The model fit with the hold- out sample was similar (RMSEA = 0.036; CFI = 0.996; TLI = 0.993), indicating the stability of the measurement model.

#### Structural model predicting PSMU and doomscrolling

**Fig. 2:**
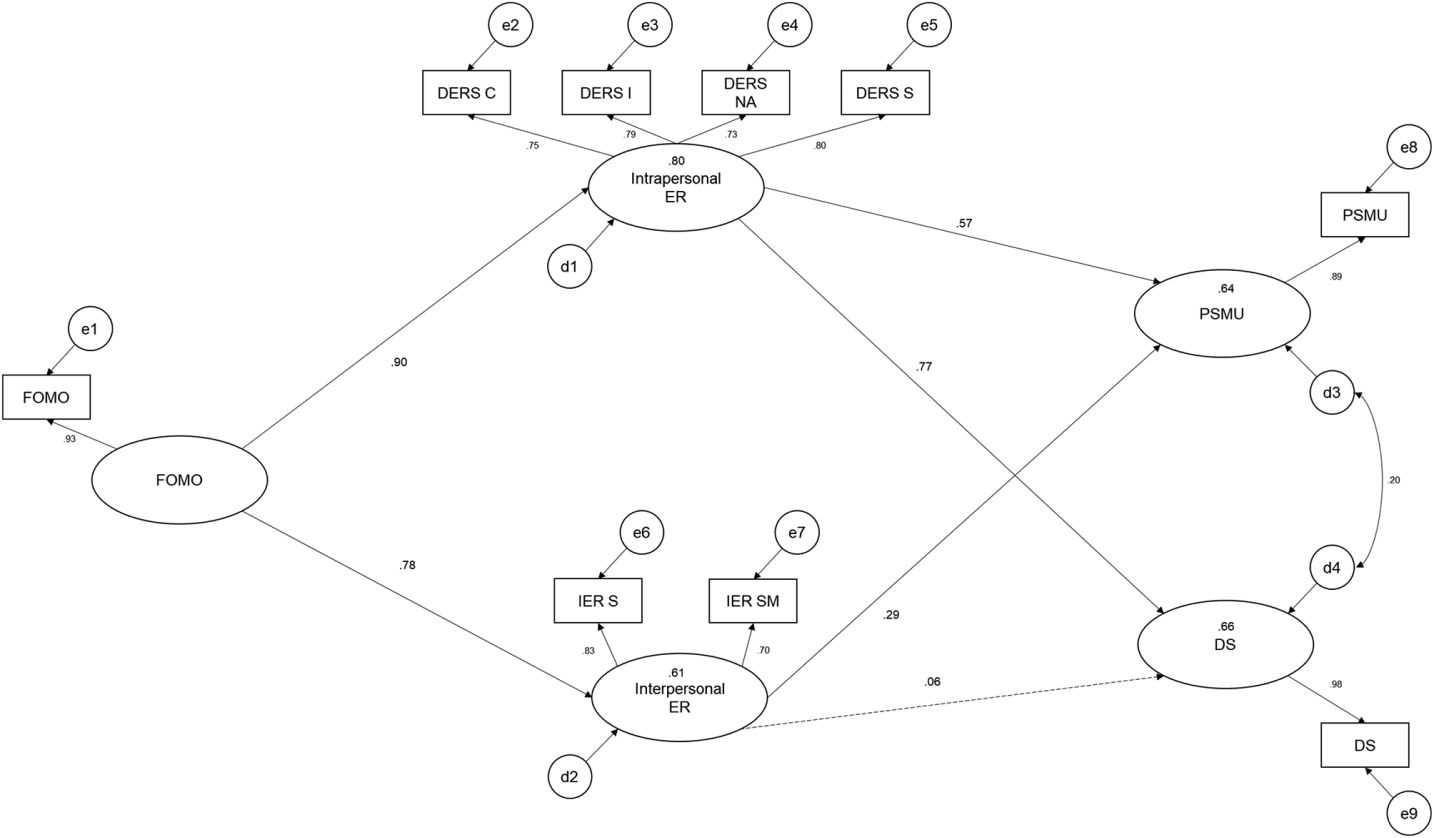
Tested parallel mediation model *Note*. ER = Emotional Regulation; FOMO = Fear of Missing Out; PSMU = Problematic Social Media Use; DS = Doomscrolling; DERS C = Clarity; DERS I = Impulse; DERS NA = nonacceptance; DERS S = strategies; IER S = Soothing; IER SM = Social Modelling. Dashed line indicates non-significance (*p* > .05)

To test the structural relationships of the proposed model, a full latent parallel mediation model was tested, with FOMO as the predictor variable, the revised DERS and IERQ modelled as parallel mediators (intrapersonal emotion regulation, and interpersonal emotion regulation), and, PSMU and Doomscrolling as the outcome variables. Both predictor and outcome variables were modelled as single-indicator latent variables. As depicted in Fig. 1, FOMO was modelled such that it predicted intrapersonal and interpersonal emotion regulation, which in turn were specified to predict PSMU and Doomscrolling. The mediation model exhibited a good fit to the data (RMSEA = 0.072; CFI = 0.972; TLI = 0.956). Taken together, the relationship between FOMO and PMSU was therefore jointly explained (fully mediated) by both forms of emotion regulation. Conversely, Doomscrolling was explained (fully mediated) by intrapersonal emotion regulation only. In the final model, the two mediators explained 64% of the variance in PSMU and 66% percent of the variance in Doomscrolling, although interpersonal emotion regulation did not significantly contribute to the prediction of Doomscrolling. Though binary sex was correlated with the outcome measures (see Supplementary Table S1), it did not impact model parameters. Figure 2 depicts the final model with standardized parameter estimates and the standardized indirect and direct effects sizes are summarized in Tables 1 and 2.

**Table 1:** Standardized direct and indirect effects on problematic social media use (PSMU)

			95% CI	
FOMO→ PMSU	*β*	*p*	Lower	Upper
Indirect effect via Intrapersonal ER	0.51	0.001	0.36	0.66
Indirect effect via Interpersonal ER	0.23	0.005	0.09	0.38
Direct effect	-	-	-	-
Total effect	0.74	0.001	0.67	0.80

*Note*. FOMO = Fear of Missing Out; ER = Emotion Regulation

**Table 2:** Standardized direct and indirect effects on doomscrolling

			95% CI	
FOMO Doomscrolling	*β*	*p*	Lower	Upper
Indirect effect via Intrapersonal ER	0.70	0.001	0.54	0.81
Indirect effect via Interpersonal ER	0.05	0.405	− 0.05	0.18
Direct effect	-	-	-	-
Total effect	0.74	0.002	0.68	0.78

*Note*. FOMO = Fear of Missing Out; ER = Emotion Regulation

## Discussion

The present study sought to contribute to the existing problematic internet use research by exploring the nature of the relationship between FOMO and problematic internet use outcomes – problematic social media use (PSMU) and doomscrolling – by including both intrapersonal and interpersonal emotion regulation as mediators. These findings add to the emerging literature on FOMO, and in particular further investigate its association with doomscrolling – an emerging element of problematic internet use behaviour. The final model revealed that while the effect of FOMO on PSMU was fully mediated by both intrapersonal and interpersonal emotion regulation, the effect of FOMO on doomscrolling was fully mediated by intrapersonal emotion regulation only. These findings suggest that although both PSMU and doomscrolling can be considered types of problematic internet use behaviours, the effect of FOMO on each can be better explained by considering difficulties in emotion regulation. Specifically, that those engaging in PSMU may fail to regulate sufficiently (i.e., intrapersonally), and potentially compensate via engaging an external entity (i.e., interpersonally); whereas, those engaging in doomscrolling may fail to regulate sufficiently (i.e. intrapersonally), potentially due to the more insular nature of doomscrolling specifically.

The results indicate that interventions that aim to reduce PSMU and may benefit from considering individual difficulties in emotion regulation, both intrapersonally and interpersonally. That is, individuals who are engaged in PSMU due to marked difficulties in managing emotional states may turn to others to compensate, and fail to develop their own intrapersonal capacity to manage distress brought about by FOMO [27]. Though social support is a notably influential correlate of mental health [35], especially relevant during COVID-19 [36], if interpersonal regulation is provided by someone online, reliance on that person to compensate for difficulties in emotion regulation may perpetuate problematic social media use. More concerning may be the emergence of close friendships exclusively in an online medium. For instance, Sheng and Kairam [37] draw from the *hyperpersonal interaction model* to explain how online platforms are a “relationship accelerator” of sorts, due to commonality in user interests, the absence of physical cues, and engagement in frequent, low-stakes interactions. Investment in these relationships is facilitated by engagement across social media and messaging platforms, which is characteristic of PSMU, and which correlates with emerging adult anxiety and depressive symptoms, and alcohol and drug use [38]. Given the results indicate a role for interpersonal regulation in accounting for the relationship between FOMO and PSMU, self-determination theory (i.e., the theoretical basis upon which FOMO was originally conceptualized) may help clarify adaptive and maladaptive outcomes of interpersonal regulation in the PSMU context. For example, Xie et al. [39] report that individuals who reported high levels of friend support, and low psychological need satisfaction, scored significantly higher on FOMO than those with high levels of both.

While the association between FOMO and doomscrolling makes conceptual sense, the measurement of FOMO may warrant further refinement in the current internet use landscape. For instance, a recent study by Zhang et al. [26] has proposed that FOMO represents a perceived threat to an individual’s self-concept, rather than from need dissatisfaction, noting that discrepancies between self-concept and behaviour generally lead to psychological distress. Specifically, FOMO may reflect an emotion comprising both *personal* and *social* elements – the former constituting the anxiety one may experience from missing out on events or opportunities, and the latter from perceptions of *how* that missing out would be perceived by a social group [26]. Future research should consider contemporary conceptualizations and measurements of FOMO that allow for some refinement of the construct, so that it is fit for purpose within the current social media environment. Such refinement will allow for greater understanding of its relationships with the broad range of problematic internet use behaviours that have emerged since its inception over ten years ago [40].

Finally, findings indicate that doomscrolling is attributable to FOMO, but more related to difficulties in emotion regulation rather than both this and interpersonal regulation. Research on doomscrolling is still in its early stages – first observed during global lockdowns due to the COVID-19 pandemic [9], indicating that it may need to be considered in relation to its place among problematic internet use behaviours moving forward. Indeed, new social media platforms emerge and take hold rapidly, while existing platforms constantly reconfigure and reinvent themselves, to capture and retain their user bases [41, 42]. Developing a better understanding of how doomscrolling relates to social media use will go a long way to helping inform the necessary interventions and clinical strategies that may reduce a general overreliance on the internet to help manage the experience of stressors or emotional states. Of similar consideration would appear to be the level of interactivity involved with engaging in certain social media and social network platforms. Some platforms (e.g., TikTok) require more passive user behaviour, which may lend themselves to doomscrolling, whereas others require more active engagement (e.g., Facebook), which may lend itself to PSMU (see 43). Such subtle differences may help contextualize the role of FOMO on emotion regulation, and in relation to its effects on PSMU and doomscrolling. Future research should therefore consider passivity as a measure alongside use and time – for instance, more passive use across more fewer platforms may be less distressing than more active use across multiple platforms. Such research may help to tailor PSMU and doomscrolling interventions to a relevant user cohort.

### Limitations and future research directions

The findings of the present study present limitations that need to be considered. Though we tested a parallel mediation model outlining plausible theoretical paths, data were cross-sectional, precluding definitive statements about causality. Indeed, relationships between FOMO and emotion regulation have been modelled differently throughout the literature (e.g., 44), noting that relationships between FOMO and problematic internet use behaviours may also be bidirectional in nature (e.g., 45) and therefore those modelled in the present study warrant further exploration. Future research should consider adopting prospective correlational or time-series designs to better establish causal links between antecedents of problematic internet use behaviours, or consequents of FOMO. In terms of our sample, it primarily comprised TikTok, Facebook, and Instagram users, meaning the profile of social media consumption may have influenced the results. Future research should consider recruiting samples balanced across their predominantly used platform, and developing multi-group models to ascertain if the associations found in the present study vary across different platforms, or usage patterns [46]. From a measurement perspective, the calibration analyses indicated that the dimensionality of emotion regulation may be worthy of further exploration within the problematic internet use context. For instance, Lavender et al. [47] have developed a four-factor measure of state-level difficulties in emotion regulation, incorporating a “modulate” factor that incorporates modulating emotions and behaviour responses, conceptually similar to *strategies*, *impulse*, and *goals* subscales from the DERS.

## Conclusion

Primarily, the research findings affirm the continued relevance of FOMO to the problematic internet use context. Emotion regulation is influential in explaining how FOMO leads to problematic internet use behaviours – however, the nature of the regulation, and its sufficiency, are important factors. While PSMU and doomscrolling can be considered facets of internet use disorder, or internet addiction, the pathways to these outcomes need to be better understood to advance knowledge and intervention in this space.

### Electronic supplementary material

Below is the link to the electronic supplementary material.

**Supplementary Material 1**: Supplementary Table S1

## Data Availability

The datasets used and/or analyzed during the current study are available from the corresponding author on reasonable request.
