# Supplementary material for: I rely on a little help from my friends: the effect of interpersonal and intrapersonal emotion regulation on the relationship between FOMO and problematic internet use
Source: BMC Psychiatry. 2024 May 24;24:384. doi: 10.1186/s12888-024-05834-9 (PMC11119391; doi:10.1186/s12888-024-05834-9)
Supplement: Supplementary file 1 — Supplementary Material 1: Supplementary Table S1 [file 12888_2024_5834_MOESM1_ESM.docx]

| Supplementary Table S1. *Correlations Between FOMO, Emotion Regulation, and Outcome Measures Included in the Study* | | | | | | | | | | | | | | | | |
| --- | --- | --- | --- | --- | --- | --- | --- | --- | --- | --- | --- | --- | --- | --- | --- | --- |
| Variable | | 1 | 2 | 3 | 4 | 5 | 6 | 7 | 8 | 9 | 10 | 11 | 12 | 13 | 14 | 15 |
| 1 | FOMO | .862 |  |  |  |  |  |  |  |  |  |  |  |  |  |  |
| 2 | DERS Aware | .251^**^ | .608 |  |  |  |  |  |  |  |  |  |  |  |  |  |
| 3 | DERS Clarity | .575^**^ | .160^**^ | .780 |  |  |  |  |  |  |  |  |  |  |  |  |
| 4 | DERS Goals | .492^**^ | .291^**^ | .411^**^ | .731 |  |  |  |  |  |  |  |  |  |  |  |
| 5 | DERS Impulse | .619^**^ | .086^*^ | .638^**^ | .490^**^ | .824 |  |  |  |  |  |  |  |  |  |  |
| 6 | DERS Non-acceptance | .627^**^ | .135^**^ | .583^**^ | .422^**^ | .573^**^ | .767 |  |  |  |  |  |  |  |  |  |
| 7 | DERS Strategies | .672^**^ | .144^**^ | .596^**^ | .518^**^ | .646^**^ | .597^**^ | .762 |  |  |  |  |  |  |  |  |
| 8 | DERS Total | .754^**^ | .381^**^ | .792^**^ | .719^**^ | .819^**^ | .777^**^ | .819^**^ | .862 |  |  |  |  |  |  |  |
| 9 | IERQ Enhancing Positive Affect | .449^**^ | .503^**^ | .251^**^ | .358^**^ | .179^**^ | .250^**^ | .264^**^ | .400^**^ | .787 |  |  |  |  |  |  |
| 10 | IERQ Perspective-taking | .598^**^ | .221^**^ | .507^**^ | .257^**^ | .582^**^ | .438^**^ | .449^**^ | .575^**^ | .424^**^ | .824 |  |  |  |  |  |
| 11 | IERQ Soothing | .607^**^ | .297^**^ | .454^**^ | .416^**^ | .499^**^ | .440^**^ | .498^**^ | .602^**^ | .534^**^ | .629^**^ | .810 |  |  |  |  |
| 12 | IERQ Social Modelling | .483^**^ | .411^**^ | .389^**^ | .370^**^ | .335^**^ | .348^**^ | .357^**^ | .502^**^ | .569^**^ | .610^**^ | .583^**^ | .776 |  |  |  |
| 13 | IERQ Total | .657^**^ | .431^**^ | .494^**^ | .426^**^ | .497^**^ | .455^**^ | .484^**^ | .639^**^ | .764^**^ | .827^**^ | .844^**^ | .835^**^ | .911 |  |  |
| 14 | PSMU | .621^**^ | .311^**^ | .526^**^ | .454^**^ | .521^**^ | .479^**^ | .562^**^ | .659^**^ | .440^**^ | .562^**^ | .471^**^ | .486^**^ | .601^**^ | .785 |  |
| 15 | DS | .727^**^ | .096^*^ | .578^**^ | .312^**^ | .633^**^ | .539^**^ | .619^**^ | .655^**^ | .245^**^ | .617^**^ | .492^**^ | .363^**^ | .534^**^ | .613^**^ | .963 |
| 16 | Gender | -.125^**^ | .047 | -.147^**^ | -.054 | -.214^**^ | -.122^**^ | -.125^**^ | -.150^**^ | -.029 | -.182^**^ | -.080 | -0.044 | -.106^*^ | -.142^**^ | -.162^**^ |
| *Note*. Cronbach’s α values are presented along the principal diagonal. ***p* < .01; **p* < .05. FOMO = Fear of Missing Out; DERS = Difficulties in Emotion Regulation Scale; IERQ = Interpersonal Emotion Regulation Questionnaire; PSMU = Problematic Social Media Use; DS = Doomscrolling | | | | | | | | | | | | | | | | |
